# Circulation of an atypical hepatitis C virus (HCV) strain at a dialysis unit in northeast India

**DOI:** 10.1002/mbo3.1147

**Published:** 2020-12-24

**Authors:** Deepjyoti Kalita, Sangeeta Deka, Kailash Chamuah

**Affiliations:** ^1^ Department of Microbiology All India Institute of Medical Sciences Rishikesh India; ^2^ Department Microbiology Gauhati Medical College Guwahati India

**Keywords:** dialysis, HCV, HCV genotypes, hepatitis C infection, maintenance hemodialysis, uncommon genotypes

## Abstract

Patients undergoing hemodialysis are at an increased risk of hepatitis C virus (HCV) infection. The implementation of standard infection control measures can substantially decrease the risk of infections and other nosocomial infections. To study the HCV infection rates and genotypes in maintenance hemodialysis subjects in a dialysis unit. A total of 196 maintenance hemodialysis subjects were tested for HCV RNA for one year at a tertiary care teaching hospital in northeast India continuously. Genotyping was performed using direct sequencing (Sanger sequencing) of the 5′UTR‐core region. The HCV infection rate was 26.0%. On phylogenetic analysis, 29 sequences clustered around genotype 3 and subtype 3f were observed. High sequence similarities (75–100% homology) were observed among the isolated sequences. High molecular similarities in the isolates from the same dialysis unit with a high infection rate (26.0%) over a relatively short period of study (10 months) indicated an ongoing nosocomial transmission. Nosocomial transmission by subtype 3f is rare, and it has not been reported in dialysis cases previously. The strain is most likely evolving from common strains such as 3b or 3i and may spread due to migration or movement of people. Urgent implementation of adequate infection control measures is required.

## Graphical abstract

A high infection rate of HCV (26%) was detected in the dialysis unit of a tertiary care hospital. All the strains of HCV were of the same genotype and subtype 3 f. This is the first time an outbreak of HCV 3f subtype is reported.

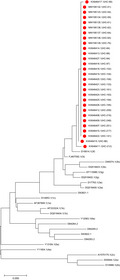

## 1 INTRODUCTION

Hepatitis C virus (HCV) causes parenteral infections, leading to complications such as chronic active hepatitis, cirrhosis, and primary liver cancer. Globally, multiple genotypes and subtypes of HCV exist owing to significant variations in the nucleotide sequence of the viral genome (Sievert et al., 2011). A patient undergoing hemodialysis is at an increased risk of HCV infection primarily due to prolonged vascular access, potential exposure to infected patients, and contaminated equipment (Sievert et al., 2011; Wigneswaran et al., 2018). In many countries, the prevalence of HCV is approximately 1–2%, and it is higher in dialysis patients (Sievert et al., 2011). A long‐term multicenter study conducted in developed countries reported an HCV prevalence of approximately 10% in dialysis patients (Jadoul et al., 2019). In less‐developed countries, the prevalence is much higher, for example, according to an Indian study, the prevalence of HCV in dialysis cases is approximately 6.7–35.6% (Jakupi et al., 2018; Roy et al., 2019). Infection rate primarily depends on factors such as infection control practices, nosocomial prevalence, HCV transmission, and the number and duration of dialysis (Wigneswaran et al., 2018). The prevalence of HCV in northeast India is higher than that in the entire nation (Phukan et al., 2003). However, the genotype distribution of HCV in northeast India has not been thoroughly investigated, and only a few studies have reported the predominant HCV genotypes 3 and 6 (Barman et al., 2018; Christdas et al., 2013; Gupta et al., 2017; Shah et al., 2016; Win et al., 2016). The infection control activities are suboptimal even in tertiary care centers. Therefore, we conducted this hospital‐based study to determine the infection rate of HCV, elucidate the co‐infection pattern with other similar viral infections such as hepatitis B virus (HBV) and human immunodeficiency virus (HIV) infections, and understand the distribution of prevalent HCV genotypes.

## 2 MATERIALS AND METHODS

**Figure 1:**
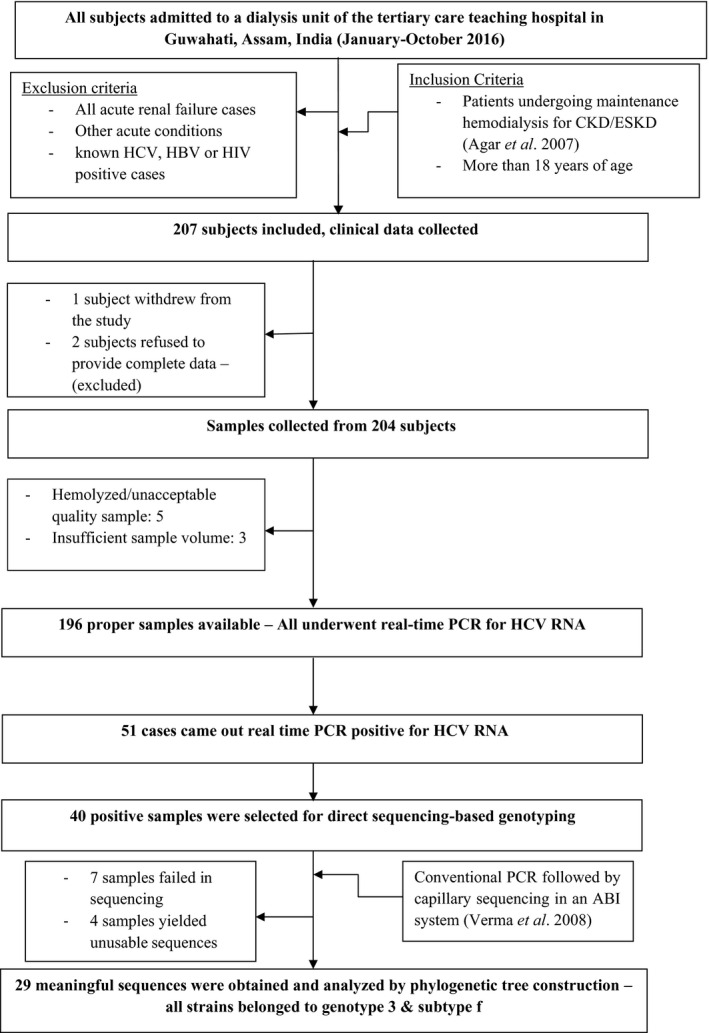
Flow diagram of the study

This cross‐sectional study was conducted between January 2016 and December 2016 (including two months of data analysis). As shown in the flow diagram (Figure 1), the inclusion criteria were as follows: 1) patients undergoing maintenance hemodialysis for chronic kidney disease (stage 5 or 4) or end‐stage kidney disease (ESKD) as defined previously (Agar et al., 2007) and 2) adults (>18 years). The exclusion criteria were as follows: 1) acute renal failure patients, 2) other acute conditions requiring dialysis, and 3) HCV‐positive or HIV‐HBV patients on initial screening at admission or anytime during the study). All samples were continuously collected.

According to the Institutional Ethics Committee (IEC) guidelines, written informed consent was obtained from all participants before initiating the study. Following pre‐test counseling, relevant clinical and epidemiological data of all participants were recorded in a standard pretested proforma. Approximately 6 mL of blood was collected from all participants, taking all necessary aseptic and antiseptic measures, and the samples were immediately dispatched to the laboratory. Subsequently, the serum was separated, aliquoted into five parts (approx. 500 µL each), and stored at −80°C. One aliquot was withdrawn, and viral RNA was extracted using the manual High Pure Viral Nucleic Acid Kit (Roche Diagnostics, Rotkreuz, Switzerland) according to the manufacturer's instructions. Extracted RNA was further processed for quantitative detection of HCV RNA by performing real‐time polymerase chain reaction (RT‐PCR) using the Cobas TaqMan 48 analyzer (Roche Diagnostics, Rotkreuz, Switzerland).

**Figure 2:**
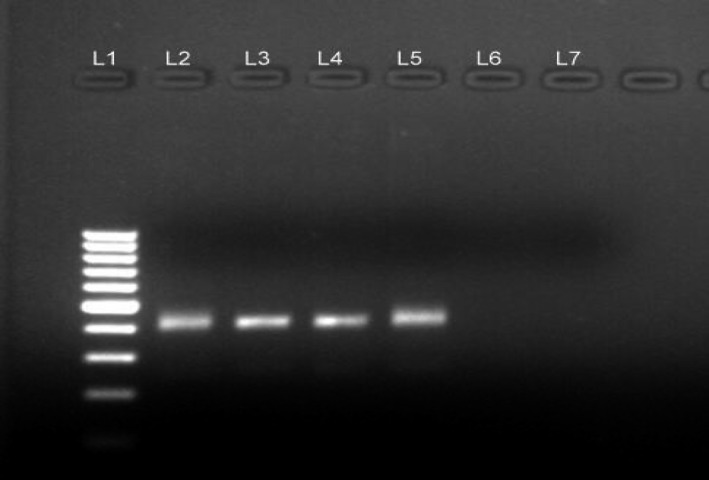
Gel diagram [L1 = 100 bp DNA Ladder, L2 = positive control (405 bp), L7 = negative control, samples L3, L4, L5 = positive for amplified product, sample L6 = no amplification]

For genotypic identification, we selected the first HCV RNA‐positive sample every week or four positive samples per month (as no positive cases were reported in some weeks), totaling 40 samples in 10 months. For initial amplification, direct sequencing was conducted to target a 405 bp region in the 5′UTR‐core region using conventional PCR (Verma & Chakravarti, 2008). RNA extraction was performed using the QIAamp Viral RNA Mini Kit (Qiagen diagnostics, Hilden, Germany), and subsequently, cDNA transcription was conducted using an outer antisense primer (IR below) using the RevertAid First Strand cDNA Synthesis Kit (Thermo Fisher Scientific, Waltham, United States) with Mo‐MLV‐Reverse transcriptase enzyme (200 U/µL) (Thermo Fisher Scientific, Waltham, United States) and RiboLock RNase inhibitor (40 U/µL) (Thermo Fisher Scientific, Waltham, United States). Subsequently, nested PCR was performed using the following primers:I‐F:ACTGCCTGATAGGGTGCTTGCGAG, I‐R:ATGTACCCCATGAG/TA/GTCGGC; II‐F:AGGTCTCGTAGACCGTGCATCATG, II‐R: CAC/TGTA/GAGGGTATCGATGAC. Amplification was confirmed after visualizing the horizontal agarose gel run (Figure 2) in a gel doc system (Geldoc XR by Bio‐Rad, Hercules, California, United States). Once confirmed, the amplified products were purified using the column‐based purification kit (Qiagen). For capillary sequencing, purified and amplified nucleotide sequences were bi‐directionally processed in an ABI PRISM 3100 automated sequencer (Applied Biosystems, Foster City, USA) along with a BigDye Terminator Cycle Sequencing Ready Reaction Kit (Thermo Fisher Scientific, Waltham, United States).

It is worth mentioning that for genotype determination of HCV direct sequencing of a conserved region (e.g., 5’UTR) followed by phylogenetic analysis (or similar) is considered a reference method (Gold standard) and it is very reproducible, hence commonly resorted to.

Sequence data obtained from the above process underwent multiple sequence alignment using BioEdit software package v.7.0.1 (freeware under open source certification). According to the Tajima‐Nei model, phylogenetic analyses were conducted using the rate heterogeneity parameter and the neighbor‐joining method. The obtained sequences were compared with reference sequences, which were downloaded from the GenBank database as previously described (Verma & Chakravarti, 2008). Confidence values were calculated using bootstrap analysis, and a consensus tree was prepared using MEGA v 3.0 (Kumar et al., 2004). To determine the percentage similarities of sequences, homology analysis was performed using the DNA Starmega Align v 5.00 software (DNA Star Inc, Madison Wisconsin, United State).

## 3 RESULTS

A total of 207 patients were enrolled; of which, 11 were rejected (baseline data is included in Table 1 while Figure 1 depicts the flow). Hence, subject enrollment was on a continuous basis, though some samples were rejected later, which was unavoidable.

**Table 1:** Baseline characteristics (n = 207).

Sr no.	Characteristics	Values
1	Total number of enrolled subjects	207
2	Rejected (hemolysis−3, inadequate specimen volume−5, incomplete data −2, voluntary withdrawal−1)	11
3	Total samples studied	196
4	Age range (mean ± standard deviation)	33–73 (51 ± 12)
5	Male:Female (171:25)	7:1

**Figure 3:**
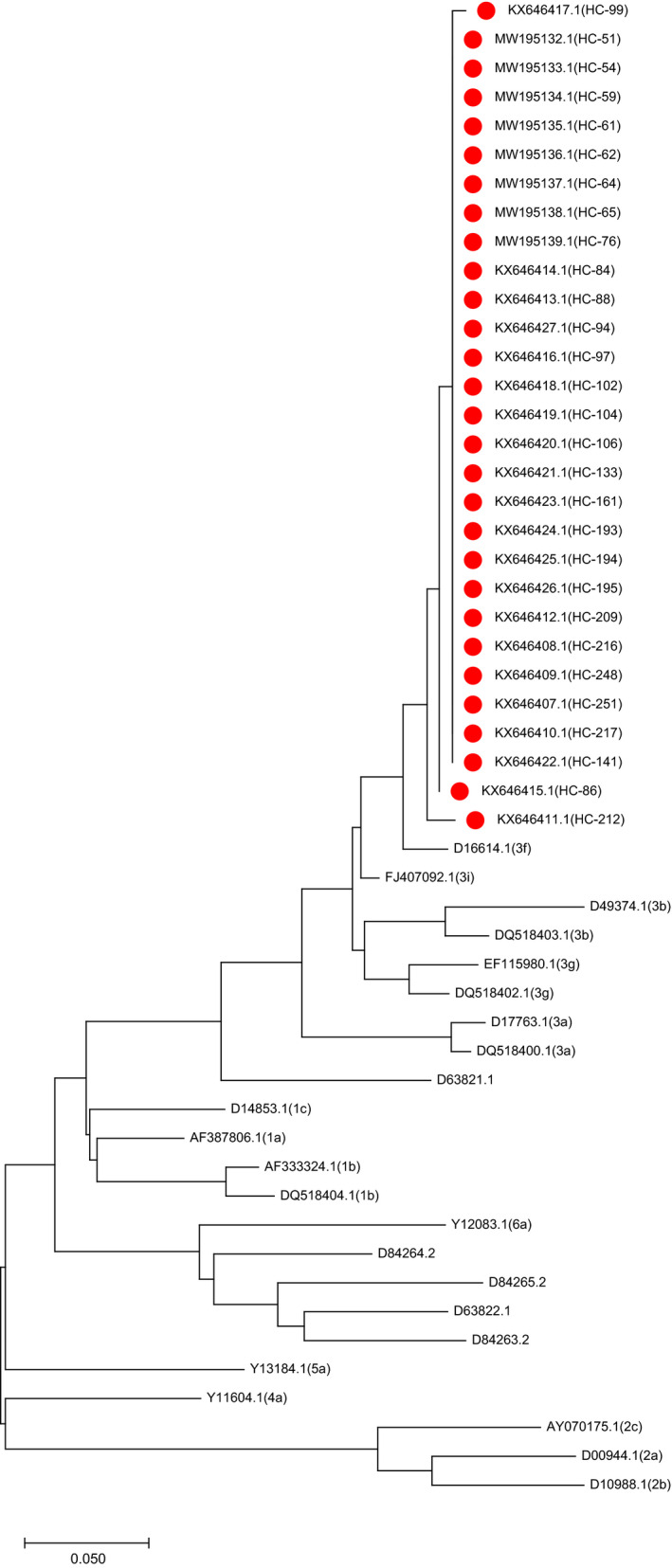
A rooted neighbor‐joining tree (for GenBank accession numbers see Data Availability Statement)

No HIV‐positive case was detected while HBV (HBsAg) and HCV‐HBV dual infections were detected in 4.8% and 1.8% of the participants, respectively (Table 2). Conventional PCR resulted in successful amplification of 33 samples, whereas no amplification was observed in seven samples (the gel diagram shown in Figure 2). On direct sequencing, 29 samples yielded useful sequence data (four samples failed or did not possess any useful sequence) that were deposited in the GenBank database (see Data Availability Statement ). Phylogenetic analyses (Figure 3) of sequence data along with downloaded sequences revealed that all 29 samples were clustered in the 3f region. Furthermore, a homology analysis demonstrated that the percent identity of sequences varied from 85.6% to 100% (https://doi.org/10.5281/zenodo.4294357).

**Table 2:** Results of testing for HCV RNA, HBsAg, and anti‐HIV antibodies in different groups.

Groups	n	Test result positive	percentage
HCV RNA test (overall)	196	51	26.0
HCV RNA test (males)	171	47	27.5
HCV RNA test (females)	25	4	16.0
HCV RNA test (in subjects up to 20 years of age)	0	0	‐
HCV RNA test (in subjects between 21 and 40 years of age)	29	5	17.2
HCV RNA test (in subjects between 41 and 60 years of age)	135	38	28.9
HIV testing	196	0	‐
HBsAg testing	196	11	5.6
HBsAg & HCV RNA positive	196	2	1.8
HCV RNA negative; HBsAg positive	196	9	4.6

Sixteen machines were present in the dialysis unit; of these, three were reserved for infectious patients (i.e., HIV‐, HCV‐, and HBV‐infected patients). No isolation policy existed for infected patients. Due to resource constraints, the dialyzers were routinely reused a maximum of 12 times. Each reused dialyzer contained the patient's name, and after each session, dialyzers were placed in separate containers labeled as clean, HCV, HBV, or HIV. A common sink was present, where dialyzers used by seronegative patients were rinsed first followed by those of HBV‐ and HCV‐infected patients. Blood samples were collected in the treatment room next to the nurses’ station. No infection control checklist and audit system to monitor the compliance of nurses with infection control protocols were visible. Alcohol‐based hand rubs were provided on request but not visible in the handwash stations. Usual hygienic precautions were observed and staff members used gloves. Gloves were changed between patients and instruction for adherence to this rule was verbal only. No written instruction or poster/display (about how and when to don/doff gloves) was available. Any mechanism was not in place to check adherence —hence, a breach in proper infection control practices could not be ruled out.

On inquiry in adjoining and other units/wards/clinical departments in the hospital, no clustering or localization of HCV cases was found. Additionally, apart from receiving treatment in the same dialysis unit, none of the positive cases was epidemiologically or family or otherwise linked. As no study on HCV infections (with genotype and subtype detection) from the locality was available, baseline data of HCV genotypes in the locality could not be found.

## 4 DISCUSSION

We observed a high infection rate and an ongoing nosocomial transmission of HCV in a busy hemodialysis unit with low priority for infection control measures.

We analyzed 196 subjects, predominantly males (M: F = 7:1) with a mean age of 51 (± 12 years). ESKD frequently develops in patients with long‐term diabetes, hypertension, and kidney diseases (Chigurupati et al., 2014; Kalantari et al., 2014; Malhotra et al., 2016). Male: Female ratio of 7:1 overwhelmingly tilted toward males despite gender not being a criterion for admission into the dialysis unit. It may be a general pattern of CRF or common predisposing factors (Diabetes etc.) here. However, no specific reason could be found for this overwhelming male preponderance. As all risk factors were equally distributed among both genders, the high number of males in the study did not affect the results. (Data not shown).

Overall, 26.0% (51/196) of the dialysis patients were HCV‐infected, and more than 90% of them were over 40 years of age (Table 2). However, in developed countries, the infection rates are considerably low (up to 10%) (Jadoul et al., 2019) as compared to those of developing countries, where the infection rates are even higher than our findings. In 2016, a study from north India reported an HCV prevalence of 33.5% in hemodialysis patients (Malhotra et al., 2016). Another study from Africa reported an HCV prevalence of 20.6% in maintenance hemodialysis patients (Halle et al., 2018). In 2019, an Indian study reported an HCV seroprevalence of 15.3% in dialysis patients (Kansay et al., 2019). APCR‐based studies from India reported an infection rate of 36.5%, whereas another similar study from Brazil reported the rate as 18.24% (Datta et al., 2015; Vidales‐Braz et al., 2015). A Kosovo‐based multicenter study reported a high HCV infection rate of 53% (Jakupi et al., 2018). Unlike the present and few previous studies, many studies employed an antibody‐based detection system such as enzyme‐linked immunosorbent assay. Antibody detection leads to low sensitivity, that is, the antibody levels in immunosuppressed patients, such as hemodialysis subjects, may not be up to the detectable levels (Sypsa et al., 2005).

The HBsAg positivity rate was 4.8% while HCV‐HBsAg co‐infection was 1.8%. No HIV‐positive subjects were observed.

A Uttarakhand (India)‐based study reported a dual infection (HBV/HCV) rate of 4.2% (Mittal et al., 2013) A Romanian study found a similar dual infection (HCV/HBV) rate of 5% (Schiller et al., 2015). Alternatively, an Iranian study reported no HIV infection in dialysis subjects (Kalantari et al., 2014). Another Punjab (India)‐based study on dialysis subjects reported the HBV and HIV infection rates as 3.06% and 1.02%, respectively (Kansay et al., 2019). HIV infection in hemodialysis subjects is rare, which is attributed to the low infection rates of HIV as compared to those of HCV or HBV and aggressive HIV screening before dialysis. HCV/HBV co‐infection is a commonly detected dual infection in hemodialysis patients, and most studies have reported its prevalence to be in the range of 1–5%, similar to our findings (Mittal et al., 2013; Schiller et al., 2015).

Phylogenetic analysis (Figure 3) revealed that all 29 sequences were clustered in the area belonging to genotype 3 and subtype 3f, suggesting a common source of infection. All samples were collected from the same hospital, and they underwent dialysis at the same unit. Homology analysis for the percent identity varied from 85.6% to 100%, which is an indication of common source transmission. A 2019 HCV outbreak in a Dutch hospital dialysis unit used the 5′UTR & NS5A region sequencing techniques to successfully determine the infection (Heikens et al., 2019). An Italian study also reported an HCV outbreak in a dialysis unit using the same method as ours, that is, direct/capillary sequencing, to determine infections due to the same genotypes (Senatore et al., 2016).

To the best of our knowledge, this is the first study reporting an HCV 3f strain‐related outbreak in our region. Reports on other HCV 3 subtypes causing chronic hepatitis or dialysis‐related infections exist; however, HCV 3f‐related outbreaks could not be retrieved from published literature. Multiple reports on the dialysis‐related transmission of HCV genotypes such as 1b, 1a, and 3a, exist (Hmaied et al., 2006; Izopet et al., 1999; Ko et al., 2020). Recently, a Pune (India)‐based study reported an HCV outbreak in a dialysis unit, where 84.6% of the isolates belonged to HCV 1b with 99.3% nucleotide sequence similarity, indicating a nosocomial transmission (Roy et al., 2019).

The genotype distribution patterns in such cases usually reflect the distribution in the general population (Ko et al., 2020). A multicenter study reported an HCV infection rate of 69% in eastern India (including our region), which was caused by genotype 3, without any subtyping data (Shah et al., 2016). The only other study on HCV genotypes from northeast India (Shillong, Meghalaya, 100 km from our hospital) reported the predominant existence of genotype 3 (48.7%) and genotype 6 (30.8%) in chronic liver disease patients (Barman et al., 2018). A Kolkata‐based study (geographically 1000 km away from our study location) predominantly found genotype 3a (56.2%) and genotype 1b(43.8%) in HCV‐infected intravenous drug users (Gupta et al., 2017). Another study from Myanmar reported a mixed distribution of HCV in high‐risk populations, mainly due to genotype 6 and genotype 3 followed by genotype 1 (Win et al., 2016). An Uttar Pradesh (a province in northern India)‐based study predominantly found genotype 3a (68.7%) and 1a (25%) in HCV patients. The other detected strains included 1b (2.9%), 3b (2.2%), 3 g (0.49%), and 3i (0.74%) (Prakash et al., 2018). The only other study reporting the detection of HCV 3f (4.44% isolates) is a New Delhi‐based study on chronic hepatitis patients, which predominantly detected 3a, 3b, and 1b strains (Chakravarti et al., 2011).

The emergence of the uncommon 3f strain as a potential outbreak agent can have other inferences. As previously mentioned, the increased incidence of HCV due to an uncommon subtype may indicate the spread and transmission due to human migration‐related infections (Gupta et al., 2017). Some studies have reported the transmission of novel HCV genotypes/subtypes due to migration (Borgia et al., 2018) According to the phylogenetic tree (Figure 3), it is obvious that the current strain is genetically related to subtype 3i and perhaps to the more commonly found 3b. Its associations with other prevalent strains such as 3a and 1b seem distant. Hence, it can be assumed that this particular strain is derived from the 3 g (or 3b) strain during transmission (Figure 3). A broader study with more subjects can provide additional clarifications.

Our study also indicates strong evidence of nosocomial patient‐to‐patient HCV transmission. Given these circumstances, the most likely cause of transmission is a breach of infection control procedures (Duong & McLaws, 2016; Jadoul, 2018). The high nucleotide similarity of the HCV strains isolated from patients indicates a common source, possibly a chronic HCV infection case.

## 5 CONCLUSIONS

The infection rate of HCV in chronic dialysis or maintenance hemodialysis subjects in a tertiary care hospital in northeast India was high (26.0%), and the co‐infection rate with HBV was 1.8%. An uncommon subtype of HCV (3f subtype) seemed to cause nosocomial transmission in the dialysis unit. Phylogenetic analyses indicated that this uncommon subtype could emerge from other subtypes such as 3 g or more common subtypes such as 3b. This new subtype may be linked to the spread of infection due to migration. Strict implementation and regular audits of standard infection control measures are urgently required (was advised to the concerned dialysis unit).

## ETHICS STATEMENT

Protocols and procedures were reviewed and approved by the Gauhati Medical College Institutional Ethics (approval no. MC/2/2015/82 dt13.5.2015). Written informed consent was obtained from each participant.

## CONFLICT OF INTEREST

None declared.

## AUTHOR CONTRIBUTION

**Deepjyoti Kalita:** Conceptualization (lead); Funding acquisition (lead); Methodology (lead); Project administration (lead); Supervision (lead); Writing‐review & editing (lead). **Sangeeta Deka:** Formal analysis (lead); Resources (lead); Validation (lead); Visualization (lead); Writing‐original draft (lead). **Kailash Chamuah:** Data curation (lead); Investigation (lead); Software (lead).

## Data Availability

Supplemental information (homology analysis of HCV sequences) is available in Zenodo at https://doi.org/10.5281/zenodo.4294357. The raw sequence data generated during the current study are available in the NCBI GenBank database, under accessions KX646407‐ KX646427: https://www.ncbi.nlm.nih.gov/popset/1219689804; and MW195132‐MW195139: https://www.ncbi.nlm.nih.gov/popset/1928718546
